# The impact of necrotizing soft tissue infections on the lives of survivors: a qualitative study

**DOI:** 10.1007/s11136-023-03371-8

**Published:** 2023-02-25

**Authors:** Jaco Suijker, Matthea Stoop, Annebeth Meij-de Vries, Anouk Pijpe, Anita Boekelaar, Marthe Egberts, Nancy Van Loey

**Affiliations:** 1 Burn Centre, Red Cross Hospital, Vondellaan 13, 1942 LE Beverwijk, The Netherlands; 2 Association of Dutch Burn Centres, Beverwijk, The Netherlands; 3 Department of Plastic, Reconstructive and Hand Surgery, Amsterdam Movement Sciences, Amsterdam UMC,, Location VUmc, Amsterdam, The Netherlands; 4 Pediatric Surgical Centre, Emma Children’s Hospital, Amsterdam UMC, Location AMC, Amsterdam, The Netherlands; 5 Department of Clinical Psychology, Utrecht University, Utrecht, The Netherlands

**Keywords:** NSTI, Necrotizing fasciitis, PICS, Quality of life

## Abstract

**Purpose:**

Necrotizing soft tissue infections (NSTI) are potentially lethal infections marked by local tissue destruction and systemic sepsis, which require aggressive treatment. Survivors often face a long recovery trajectory. This study was initiated to increase understanding of the long-term impact of NSTI on health related quality of life (HRQoL), and how care may be improved.

**Methods:**

Thematic analysis was applied to qualitative data from 25 NSTI-survivors obtained through two focus groups (*n* = 14) and semi-structured interviews (*n* = 11).

**Results:**

The median age of the participants was 49 years, 14 were female. The median time since diagnosis was 5 years. Initial misdiagnosis was common, causing delay to treatment. Survivors experienced long-term physical consequences (scarring, cognitive impairment, fatigue, sleeping problems, recurrent infections), psychological consequences (traumatic stress symptoms, fear of relapse, adjusting to an altered appearance, sexual issues) and social and relational consequences (changes in social contacts, a lack of understanding). The disease also had a major psychological impact on family members, as well as major financial impact in some. There was a strong desire to reflect on ‘mistakes’ in case of initial misdiagnosis. To improve care, patient and family centered care, smooth transitions after discharge, and the availability of understandable information were deemed important.

**Conclusion:**

This study reveals that NSTI have a large impact on physical and psychosocial wellbeing of survivors and their relatives. Except for a few differences (misdiagnosis, fear for re-infection and actual re-infection), the patient experience of patients with NSTI is largely similar to those of burn survivors. Thus, questionnaires to assess HRQoL in burn survivors may be used in future NSTI studies.

## Background

Necrotizing soft-tissue infections (NSTIs) are rare, fulminant infections marked by severe tissue destruction and systemic toxicity, with a mortality rate of 10–32% [1–7]. Quick recognition is essential, followed by multi-disciplinary treatment which includes broad-spectrum antibiotics, debridement of necrotic tissue, and -if needed- resuscitation and organ support [8, 9]. Patients are regularly admitted to burn centers because the large wounds require specialist care. Those who survive the acute phase face a long recovery process, both within the hospital (wound care, reconstructive surgeries, regaining mobility) and after discharge (rehabilitation, outpatient visits, treatment of scar related issues), a process similar to that of burn survivors [10, 11]. Even with optimal care, survivors’ health-related quality of life (HRQoL) may be affected by lasting physical consequences, which include scars, decreased energy level and occasionally (± 10%) amputation, as well as psychosocial consequences such as posttraumatic and depressive symptoms [12–19]. Prospective studies predominantly using the SF-36 to investigate HRQoL demonstrated reduced physical and mental health, and dysfunction with appearance [13, 20], although other studies could not establish lower mental health functioning [12, 14].

Studies using generic HRQoL questionnaires may not capture the full impact of this rare disease for which qualitative designs are better suited. In recent years, several qualitative studies have been performed addressing early disease signs, the stages of the disease and the impact on family members [21–23]. However, qualitative studies documenting the impact on long-term HRQoL outcomes (> 1 years after the initial disease) are still limited [4]. Previous qualitative studies revealed new findings (e.g., fear of infection) not included in quantitative studies, and provided an in-depth understanding of the affected domains, indicating room for qualitative studies that can broaden our understanding of the long-term consequences of NSTI [19, 22]. Moreover, with a changing paradigm in health care, induced by increasing access to medical information, a patient-clinician partnership relation and focus on patient-centered care, studies in which the patient’ perspective is considered gain importance [24].

Therefore, the aim of this study is to explore experiences and perspectives of NSTI survivors at least 6 months after the diagnosis to better understand the long-term impact of the disease, how treatment may be improved, and which HRQoL domains may be of interest for future studies.

## Methods

A qualitative study was performed in NSTI-survivors using focus groups and interviews. The consolidated criteria for reporting qualitative research (COREQ) guideline was used to ensure proper reporting of methods, results, and discussion [25].

### Recruitment

The study used convenience sampling, including adult (> 18 years) NSTI survivors at least six months after the diagnosis to be able to reflect on the course of the disease and its longer term consequences. Exclusion criteria were insufficient proficiency of the Dutch language. Participants were recruited through posting the study information by a patient-representative in the NSTI survivor Facebook group counting 75 members. Fourteen NSTI survivors (including one colleague who survived NSTI and was recruited directly) participated in the focus groups, which took place in November 2018. One of the participants who was unable to join the focus groups, participated in an individual interview. The other interviewees were recruited during a peer support meeting for NSTI survivors, or at the outpatient departments of two affiliated burn centers, resulting in six and four inclusions, respectively. The eleven face-to-face interviews took place between July 2019 and June 2020.

### Data collection

A semi-structured approach was used. The interview guide for the focus groups was based on a previous qualitative study in burn survivors, since the consequences of NSTI as well as the required care bears many similarities with the care for burn victims, also including intensive care syndrome (PICS) [26, 27]. The topic guides were divided into seven sections (disease course, rehabilitation, scars, family impact, vocational impact, intimacy impact, positive changes). Questions posed were open-ended, and prompts were used to clarify and check the answers. The focus groups were led by a male medical doctor (JS), and a female psychological researcher (ME, PhD). Interviews were conducted by a male medical doctor (JS) as part of his doctoral thesis, a female research nurse (MS), and a female senior researcher with a background in nursing and psychology (NVL, PhD). ME and NVL were trained in qualitative research at Utrecht University, and are experienced qualitative researchers [26, 28–32]. JS and MS were trained by NVL and ME before the interviews, and supervised by them during the first interviews. The two focus groups, and nine interviews were conducted face-to-face at the hospital or a research institute. One interview was conducted at the patient’s home and one online. The focus groups and interviews were recorded and transcribed verbatim by a transcription service. The focus groups took 133 and 102 min, respectively, the mean duration of the interview was 70 min (range 50 to 115).

### Data analysis

Thematic analysis was used to analyze data that allows gaining insight into the participant’s subjective account of their experiences. The systematic process of constant comparison was applied during the study, i.e., information from new interviews was compared with existing codes to identify similarities and differences [33]. We started with an inductive open coding process, in which the interviews were read line-by-line and fragments were extracted and assigned a code that summarized the meaning of the fragment. Subsequently, axial and selective coding was applied, which resulted in topics organized in different domains. The data were analyzed in MAXQDA 2018.

Triangulation of data was performed by three independent researchers with different backgrounds (JS, MS, NVL). The two focus groups were coded independently by JS and ME, the first five interviews by JS, MS and NVL, and subsequently themes were compared and discussed. Subsequent interviews were coded by JS and NVL, and interviewing was continued until saturation was achieved. Themes were inductively derived which allows to identify personal reflections on the patient journey. Quotations that clearly represented the derived themes were selected. The results were discussed with a patient-representative to enhance the trustworthiness of the data and the relevance for the patient group as a whole, leading to some changes in the description of subthemes. Semi-quantification was used to express relative importance of terms [34]. A ‘few’ was defined as two or three, ‘several’ as four to seven, ‘many’ as eight or more, and ‘nearly all’ as more than 20.

## Results

**Fig. 1:**
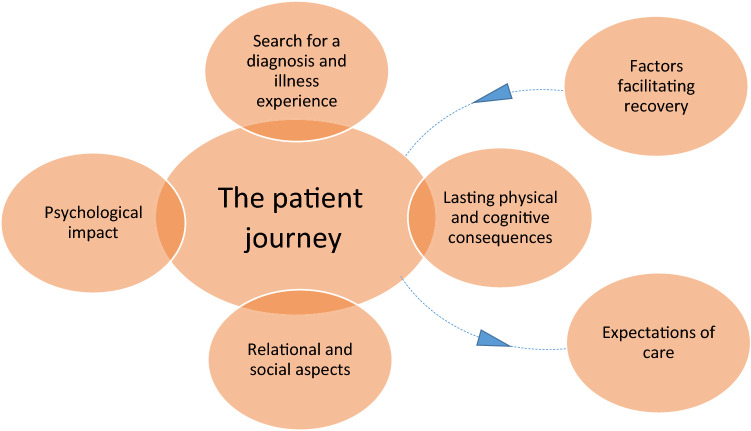
The six main themes identified in this study. Of these, four form ‘the patient experience’. ‘Factors facilitating recovery’ influence the patient experience, and ‘expectations of care’ are ideas and suggestions formed based on the past and current patient experience

**Fig. 2:**
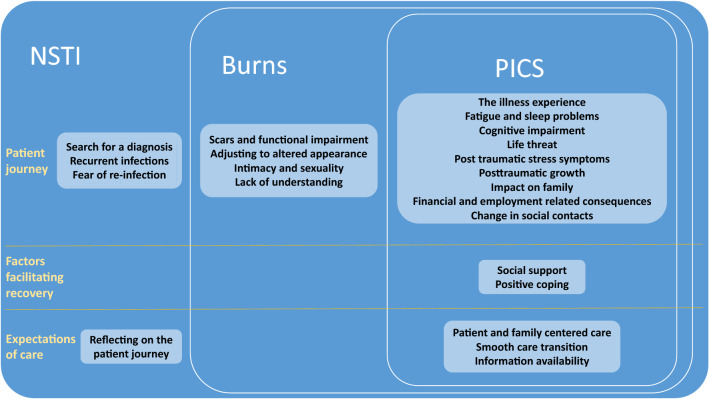
The identified themes in comparison with those identified in burn survivors and patients with PICS. While NSTI survivors face unique problems, there is a large overlap with problems encountered in burn survivors and patients with PICS. A unique recommendation is the desire to reflect on the patient journey, overlap with burns and PICS is seen in care transitions, patient and family centered care, and desire for information

The baseline characteristics of the 25 study participants are displayed in Table 1. The median age was 49 years, most (*n* = 14) were female. The time since diagnosis varied between 6 months and 17 years. All had skin defects, of which the majority (*n* = 21) required skin transplants. Most participants were employed (*n* = 23), had a partner (*n* = 21 and/or children (*n* = 20) at the time of diagnosis. Thematic analysis revealed a total of six themes and twenty subthemes, as displayed in Table 2*,* in which illustrative quotations for each subtheme are also displayed. Four themes formed ‘the patient experience’, as graphically displayed in Fig. 1*,* while two other themes either influenced this patient experience (factors facilitating recovery), or were a result of the patient journey (expectations of care). In Fig. 2*,* the overlap of themes with those previously described in burn victims and patients with PICS is shown.

**Table 1:** Characteristics of study participants participating in the focus groups (n = 14) or individual interviews (n = 11)

	All (*n* = 25)	Focus groups (*n* = 14)	Interviews (*n* = 11)
Age (years), median (IQR) (min–max)	49 (44–56) (29–64)	50 (46–57) (41–62)	49 (38–55) (29–64)
Female, n (%)	14	8	6
Comorbidities*, n (%)			
Any	6	5	1
Immunocompromised	3	3	0
Cardiovascular	2	1	1
Diabetes mellitus	1	1	0
Location(s) affected			
Head or neck area	1	0	1
Arm(s) or thoracal	10	7	3
Anogenital, abdominal or gluteal	10	6	4
Leg(s)	14	6	8
Time since diagnosis (years), median (IQR) (min–max)	5 (2–8) (1–17)	5 (3–9) (1–17)	3 (1–7) (1–15)
Start symptoms to diagnosis (days), median (IQR) (min–max)	4 (2–7) (1–30)	4 (2–6) (1–12)	4 (2–7) (1–30)
Estimated area grafted (TBSA), median (IQR) (min–max)	4 (2–7) (0–16)	5 (4–7) (0–16)	3 (0–7) (0–12)
Highest education**, *n* (%)	*N* = 1 missing	*N* = 1 missing	
ISCED level 3	1	0	1
ISCED level 4–5	13	7	6
ISCED level 6–7	9	5	4
ISCED level 8	1	1	0
Employment type at diagnosis	*N* = 1 missing	*N* = 1 missing	
Unemployed	1	0	1
Fulltime employed	15	9	5
Parttime employed (< 32 h/week)	8	3	5
Partner at time of diagnosis, *n* (%) yes	21	12	9
Children at time of diagnosis, *n* (%) yes	20	12	8

*IQR* interquartile range, *min* minimum, *max* maximum, *ISCED* international standard classification of education

*Data on active malignancy, and respiratory and renal comorbidity were also collected, but present in none of the participants

**Classified according to the International Standard Classification of Education 2011, http://uis.unesco.org/sites/default/files/documents/international-standard-classification-of-education-isced-2011-en.pdf

**Table 2:** Identified themes and subthemes with their descriptions and illustrative quotes based on focus groups and interviews with a total of 25 survivors of NSTI. Codes of participants to focus groups (FG) and interviews (INT) are provided with quotations

Theme	Subtheme	Description
The search for a diagnosis and illness experience	Search for a diagnosis	The course of symptom development, usually with a specific symptoms (flu-like symptoms, swelling, redness, pain) which in many led to delayed recognition and misdiagnosis, all while feeling something was wrong *They don’t understand, of course. You don’t know what caused it. My arm was two centimeters wider than the other one, but my blood pressure was real low and he [the family doctor] says is your blood pressure always that low? I say no, I gave blood only last Thursday and everything was fine, because otherwise you can’t give blood. He says OK, but I’m going to send you home with some painkillers. (FG1)*
	The illness experience	A physically and mentally intensive treatment period which included multiple (emergency) surgeries, respiratory support and delirium on the ICU, (painful) wound treatment, reconstructive surgeries *They changed it [the dressing] and that was excruciating. You’re better off giving birth to three children[…] I screamed so much. (FG12)*
Lasting physical and cognitive consequences	Scars and functional impairment	Longtime scar related issues including functional limitations, tight feeling, edema in extremities, and pain to touch *And certainly when I’m at the physiotherapist’s. I had a lot of nerve pain in the beginning, from my knee all the way down to my ankle. (INT7)*
	Fatigue and sleep problems	Varying degrees of physical and mental fatigue, leading to decreased capacity to participate in tasks, work and social events, sometimes associated with insomnia *And in daily life you do have certain limitations or things you can’t do anymore, or you get tired easily, like I just said. […] You do have to deprive yourself of a lot. (FG10)*
	Cognitive impairment	Decreased concentration, ‘brain fog’, forgetfulness, poor planning, clumsiness, difficulty finding words and difficulties multitasking *Then I was driving the car and then I’d think: oh right, the windshield wipers need to go on. And then: shoot, how do I do that. I’d just forgotten. (INT5)*
	Recurrent infections	Recurrent severe non-necrotizing infection(s) in a few, requiring hospital admission and sometimes surgical exploration *So I’ve been admitted to hospital three times again in the past three years with cellulitis. And then you do get similar symptoms again. (FG3)*
Psychological impact	Life threat and traumatic stress symptoms	Processing of life threat leading to various emotions (anger, helplessness, grief), and the persistence of hypervigilance upon fever, hospital visits, or sounds similar to ICU apparatus, leading to fear *As soon as I get a fever or feel a little feverish, I panic. & I’ve now had trauma therapy one time. Currently we are talking about the moment it became clear I needed to go for surgery (INT2)*
	Adjusting to an altered appearance	Being ashamed for scars or gained weight, and shame for not being able to function like others, reinforced by looks or comments of strangers, leading to changes in dress and avoiding certain situations, and the need for acceptance *And people stare too. […] I always came home crying after I’d been to the swimming pool. (INT3)*
	Posttraumatic growth	Valuing life (almost) as much as before the disease despite limitations, due to appreciating ‘the small things in life’ more *But on the whole, I am a happy person now and I’m OK about what happened to me. I think I live life with more awareness now. (FG5)*
	Intimacy and sexuality	Decreased sexuality in some due to a combination of physical (pain upon touch, scarred genitals) and changed appearance by the patient, as well as due to partners feeling less attracted *Do you think my wife was interested in sex right away? Well, no way. I’m glad I’m back home again and can sleep next to her. I say, whatever happens in the future, we’ll deal with it then. (FG2)*
Relational and social aspects	Impact on family	Partner becomes caregiver, emotional impact on the partner, children and other relatives, behavioral and emotional problems in children, and shame for the altered appearance of the patient by family and children *Every time I took her [daughter] to school, there were tears […] so that was when we started taking her to trauma therapy. (FG1)*
	change in social contacts	Various positive (growing closer to partner and some friends) or negative (losing partner, losing contact with siblings, losing friends) changes in social dynamics *I’ve lost nearly all my friends, only one left (FG14)*
	Lack of understanding related to the disease	Misconceptions among family, peers and employers about the cause of the disease (stigma), no understanding of the severity of the disease and especially not the long-term consequences, or a continuous focus on the disease instead of the patient itself *It’s unhygienic, as if it’s got to do with hygiene. That’s what a lot of people think. (INT1)*
	Financial and employment related consequences	Depending on previous employment and financial status, varying degrees of financial impact (up to bankruptcy) and work-related problems (up to complete inability to work anymore) could have a significant impact of the life and future of patients and their family *We’ve had to drastically change our future plans. We sometimes go overdrawn, but we try not to let our kids notice. That is an extra worry for us. (INT5)*
Factors facilitating recovery	Social support	Practical and emotional support of the partner, family and friends were mentioned by many as essential during the disease and recovery process *That also made me strong. The moments you feel real bad, and your friends are just there for you. Just: “Come on, we’re going to the store”, or “I’ll just do some laundry for you” (INT3)*
	Positive coping during recovery	Seeing things in perspective, focussing on when can instead of what cannot be done, being open to others, and taking back control were among the various positive coping strategies mentioned as helpful *Focusing on what you can do and not on what you can’t do. (FG12)*
Expectations of care	Reflecting on the patient’s journey	The desire to reflect on (mistakes made during) the early disease course by preference with their initial health care professionals followed by appropriate policy changes to prevent repetition of the mistake if applicable *The clinic started up a whole procedure themselves. Did we do something wrong? Should we have noticed? Well that’s certainly an error they made. Because, as he said himself, I no longer let anyone be assessed over the phone. I also think that shouldn’t be allowed (INT1)*
	Patient and family centered care	Patients and their families desire to be heard, be involved in decisions, and have their psychological needs met by health care professionals *[interviewer:] And can you tell me more about that, what’s bad? [respondent:] […]Communication wasn’t good. It’s such a big hospital, I’m just, you’re lying there but, you’re just a number. (INT11)*
	Smooth care transition	Smooth transition of care involving multiple relevant caretakers to aid on different aspects of physical and mental consequences of the disease *Personally I think the aftercare, that could have been a lot better. […] That it was already arranged by the hospital […] It was just really tiring. And then we also called the doctor to say we needed dressings, home care needed to be set up, because I basically needed help. (INT2)*
	Information availability	The availability of reliable information (from health care professionals and/or online) to patients and their families was considered important, and currently often insufficient. A clear exception was information regarding the prognosis in the acute phase, which according to many would have demotivated them *So I just started searching the internet myself, just looking- Because I had no idea what I’d had. Bits and pieces, and it was much later when they told me that, but yes I really did have to look it up myself. (FG7)*

### The search for a diagnosis and illness experience

The ***search for a diagnosis*** was marked by the onset of mostly non-specific symptoms (pain, redness, swelling, flu-like symptoms, altered mental state (brain fog), diarrhea) while NSTI survivors often had the feeling something was profoundly wrong. In many patients, the symptoms were misinterpreted by the general practitioner (GP) or in the emergency department (ED) as a simple skin infection, venous thrombosis, arthritis, food poisoning or flu, after which many were sent back home. There was substantial variation in time from onset of symptoms to diagnosis (hours to weeks). When NSTI was ultimately suspected, many experienced severe life-threat, although several others were too gravely ill to comprehend the severity of the situation.

***The illness experience***, as one patient phrased it, could be described as a rollercoaster. The (suspicion of the) diagnosis NSTI led to the initiation of an array of (often invasive) treatment modalities including (multiple) surgeries, admission to the intensive care unit (ICU) and respiratory support. Many had significant memory gaps of the first days (sometimes weeks) of hospital admission, as well as fearful and unreal (hallucinatory) memories of their time in the ICU, often related to delirium. Many only realized the life threat after regaining consciousness, which often elicited panic, fear, helplessness and insecurity. Seeing the (extensive) wound for the first time was described as ‘horrific’, although several described it as ‘not so bad’. A few expressed extreme wound pain during dressing changes partly attributed to health care professionals’ limited experience with wound care in general hospitals. In many, multiple reconstructive surgeries including skin transplantations were needed, which could cause severe pain due to tearing of the newly ingrown skin transplants upon movement.

### Lasting physical and cognitive consequences

***Scars and functional impairment*** were, in varying degrees, present in all. Scars were less elastic compared to normal skin, and the feeling of contraction was described as a ‘corset’ by one. Sensory problems included pain, often tingling or shooting pain, as well as burning sensations and (sometimes severe) itching. The decreased range of motion in affected limbs could hamper (intensive) sports and limit walking distance. Lymphedema in the affected extremity was mentioned as a major issue in several patients, for which some received therapy for years. Many patients underwent multiple scar-revision surgeries over the course of many years, up to a dozen in one.

***Fatigue and sleep problems*** were reported by nearly all in the short-term, and in many these problems persisted in the long-term. It could severely limit the ability to perform regular daytime activities and limiting social activities (for example (birthday) parties). This required some to be selective in which activities they attended, and led to an increased focus on quality of social relationships and one-on-one interactions. In some, fatigue seemed to be caused by insomnia, related to the inability to find a good sleeping position due to sensitive scars, or related to psychological problems. This led to decreased energy during the day and daytime sleepiness.

***Cognitive impairment*** was reported in many. Impaired cognitive domains included memory, attention and concentration, word finding, and executive functioning such as planning, organization and decision-making. People also reported an altered cognitive state or brain fog, as well as apraxia in some leading to clumsiness while paying or difficulties while driving. The impact of cognitive problems on daily life varied from minor to severely limiting their ability to work and participate in social life.

***Recurrent infections*** affected the lives of a few survivors. While the infections proved to be non-necrotic, sometimes hospitalization during multiple days was needed. The confrontation with new infections elicited fear of recurrence of NSTI.

### Psychological impact

***Life threat and traumatic stress symptoms*** were reported by many. Survivors realized they had been in a life-threatening situation and/or at risk for amputation of extremities, which was associated with feelings of helplessness and loss of control. A mixture of other feelings like anxiety, panic, insecurity, bewilderment, confusion, anger, sadness and loneliness were reported. In the longer term, many suffered from posttraumatic stress symptoms, hypervigilance and anxiety that was particularly related to medical events. Fever and pain could elicit feelings of anxiety that the disease may re-occur, and hearing sirens or drawing blood could elicit flashbacks and lead to panic attacks. Several received professional psychological support, to come to terms with what happened, or received treatment for posttraumatic stress symptoms. Existential questions such as ‘why me?’ were also expressed.

***Adjusting to an altered appearance*** was another major psychological theme in the aftermath of NSTI. Early in the process, it was hard to accept the altered appearance. One participant even experienced disembodiment and called her leg “horror leg”. On the long-term, many were hesitant to show their scars and tried to cover the scars with clothes, for example wearing long pants in the summer. In some situations, e.g., visiting a swimming pool, they felt self-conscious because of the scarring, and staring by strangers led some to avoid swimming pools. In addition, family members’ reactions could be embarrassing, such as when a survivors was asked to cover her scars. A few women expressed feeling less ‘feminine’, although this improved over the years.

***Posttraumatic growth*** was also reported. Many survivors expressed gratitude for all that they had, and valued ‘the small things in life’ more. Some said they were as happy as before the disease, despite obvious long-term consequences.

***Intimacy and sexuality*** were affected in several patients. Most reported problems were associated with touching sensitive scars, making it difficult to engage in physical contact, or needing time to adjust to the changed appearance.

### Relational and social aspects

***Impact on family*** (partner, children, parents, other relatives) was extensive in nearly all. Practically, role shifting took place by partners who had to take over care for children and/or household tasks. In one case, a partner had to quit their job. Some described their partner got into the ‘survival mode’. Emotionally, the family consciously experienced the battle for survival, in contrast to the patient, which was highly distressing. For those with young children, dilemmas emerged about what information should be provided to the child, and whether or not the child should visit the parent in the ICU. Even years later, the patient’s partner, children, parents and siblings could get very emotional when discussing the disease. A few patients’ children, as well as a sibling, received trauma therapy related to NSTI. Some children developed separation anxiety, or expressed fear of recurrence of the disease when patients needed to see a doctor.

A ***change in social contacts*** occurred in many, either positive or negative. As one phrased it, the relation with the partner ‘became more intense’. In contrast, another reported losing a partner unable to adapt to the changed appearance or changed relational dynamics. Friendships could become closer, but losing contact with friends or family was reported as well. According to several patients, the disease and its aftermath revealed one’s true friends. While this could initially be painful, losing non-committed friends was ultimately viewed as a positive change.

A ***lack of understanding related to the disease*** by others was mentioned by several patients. This included believing the disease was caused by poor hygiene, as well as a persistent fear for contamination, in one case expressed by an occupational therapist. Peers, as well as employers, could often not grasp that closure of the wound did not mean full recovery, leading to misunderstanding when patients were unable to work at a similar level as before the disease. In general, the social environment did not always understand the impact of the disease, which could lead to disturbed relationships.

***Financial and employment related consequences*** of the disease depended partly on pre-disease employment and financial status. In several patients, the impact of the disease on financial security and employment was substantial (e.g., bankruptcy). Several were unable to return to work, whereas others got back to work within a few months. In those less fortunate, the consequences of the disease had an extensive impact on the patients and their families, with one expressing feeling guilty towards the children for this. Financial support from social security was deemed insufficient by those who were severely financially impacted.

### Factors facilitating recovery

***Social support***, both by a partner, family, or friends, was mentioned as an essential part in recovery. This included both emotional support (ability to discuss problems, being present), or instrumental support. The degree in which support was delivered by a partner, family or friends, differed considerably across patients.

***Positive coping during recovery*** included positive refocusing, putting things into perspective, and taking responsibility for one’s recovery. Being active (i.e., sporting) was also considered beneficial. Also, taking the day as it comes was mentioned as helpful. Not covering scars and being open to others about what happened facilitated normalization. In coming to terms with the event, keeping a diary was considered helpful by a few. One patient mentioned a photoshoot in which scars were visible helped her to overcome shame. Another visited the ICU because of recurrent, unrealistic, frightening memories of the period there. Meaning making could also be found in more spiritual ways, for example by believing the disease was not a coincidence, and a higher power was with them along the journey.

### Expectations of care

***Reflecting on the patient’s journey*** was felt important. Many participants struggled with feelings of anger resulting from the misdiagnosis by GP or specialist. Particularly when the physical examination had been brief, they felt not taken seriously. Participants appreciated interest from the GP in their situation and valued a hospital visit. Participants felt the need to reflect on the events preceding their diagnosis with the involved clinician(s) and would appreciate apologies when they were misdiagnosed. In that case, the hope that clinicians would learn from their case was expressed. One patient said, currently being aware of the rareness of the disease, the anger towards her GP may not have been fully justified.

***Patient and family centered care*** was deemed important throughout the treatment process. Patients wanted to be ‘seen’, and together with their families being involved in treatment decisions. They also expected health care providers to care about their psychological needs, and those of their families, and to actively assess those and initiate help or treatment when needed.

***Smooth care transition*** was hampered in several ways. Aftercare related to, e.g., wound care at home was not well taken care of and participants struggled to find adequate help. Some participants regretted not having been treated in a rehabilitation center and suggested this should be offered as standard care. They felt that the multidisciplinary treatment in rehabilitation centers helps speed recovery. Offering contact with peer NSTI survivors was recommended, although not all were necessarily interested in this.

***Information availability*** was mentioned as important to both patients and their family, both in the acute setting, as well after recovery. The information provided by medical professionals, as well as online information, was found unsatisfactory. Reliable and understandable online information was felt helpful to patients and family, and may aid in reducing stigma. A notable exception regarding information was sharing the prognosis with patients in the acute phase, which most felt would have demotivated them and could have led to them giving up.

## Discussion

This study confirms that surviving NSTI can have a significant impact on long-term HRQoL The study showed a large overlap with HRQoL problems reported after burns and PICS [10, 11], of which some were not previously described as problems after NSTI [19, 22]. Among these are ‘fatigue and sleeping problems’ and ‘cognitive changes’, which showed to profoundly impact the lives of survivors. Another novel finding was the actual recurrence of severe (non-necrotizing) soft tissue infections requiring re-admission. This study also yields directions for how to improve care from the patient perspective, which included the need to reflect on mistakes (misdiagnosis) made upon initial presentation, patient and family centered care, care transition and information.

The long-term impact of NSTI on HRQoL relates to the local and systemic effects of the disease. The local, destructive effects of the disease and its surgical management often lead to extensive wounds similar to deep burns, which require similar surgical interventions and wound care. The systemic effects, resulting from sepsis as well as prolonged ICU admission, may cause post intensive care syndrome (PICS), which is a syndrome of various symptoms experienced in patients after ICU admission, including weakness, fatigue, sleep disturbances, and decreased cognitive performance (memory disturbance, slow processing and concentration), as well as posttraumatic stress symptoms, anxiety, depression, and sexual dysfunction [27, 35]. These local and systemic effects corroborate a previous study among NSTI survivors [19]. Additionally, our study revealed that ‘fatigue and sleeping problems’ and ‘cognitive changes’, were long-term problems affecting HRQoL, problems also present after burns, which adds to the understanding of the impact of NSTI on patients’ lives [36, 37].

Beyond the similarities with burns and PICS, this study revealed unique health-related problems with specific long-term consequences, as visualized in Fig. 2. The search for a diagnosis, which usually starts with flu-like symptoms followed by the feeling ‘something is clearly wrong’ leading to a misdiagnosis, seems a typical NSTI-related issue [21]. In line with previous reports, where it was reported that patients were sent home or sometimes an ambulance was denied, this study confirms many NSTI survivors were sent home [21, 38]. The misdiagnosis and prolonged period of uncertainly elicits distress and this study adds that it leads to the need to reflect on their illness experience with health care providers who were involved in the initial diagnostic process. Another finding unique to NSTI is fear of recurrence. This has been especially well described in NSTI research among both patients and their families, and exists throughout all phases of the patient journey [15, 19, 22, 23]. While this fear may be unsubstantiated for most participants, a few indeed experienced re-infections that could lead to hospitalization and surgical inspection, which was very distressing to patients and their families. More research is warranted to examine to what extent re-infections occur after NSTI.

This study provides directions for improving care from the NTSI survivor’s perspective. As indicated, one unique aspects relates to ‘reflecting on the patient’s journey’, which entails the desire to reflect on the initial diagnostic phase, resulting from the misdiagnosis and the feeling that mistakes were made. The underlying need to reflect on their illness experience was twofold: it helps to come to terms with what happened, and health care professionals should learn from their story to better help future patients. In line with other medical populations, there is a need to optimize ‘patient and family centered care’, in which both patients and family members are part of the care process [39], there is a need for a smoother care transition in line with previous findings [22] and ‘information availability’ should be improved [40].

This study confirms the extensive impact this disease can have on survivors’ families, including symptoms of posttraumatic stress in partners, children and other family members, and separation anxiety and behavioral problems in children. In line with burns and PICS literature, families may also experience an array of psychological symptoms [41, 42]. Davidson and colleagues advocate that open communication and the inclusion of family members in the decision-making process may help family members cope with the consequences of the disease. This corresponds with findings in this study, in which patients expressed a need for them and their families to be included in the decision-making process and treatment plan. Family centered care in which the patient and family are actively involved in the decision-making process, should be pursued to increase care quality and satisfaction with treatment.

We believe the current findings have both clinical and research implications. Clinically, the large impact of NSTI on patients and their families emphasizes the need for multi-disciplinary treatment in a specialized care center. Given the many similarities between the treatment and long-term HRQoL problems between patients with NSTI and burns, we believe burn centers are well equipped to provide such specialized care, especially in patients with bigger or more complex wounds. As scars were an identified theme in this study, the impact of reduction of scar size on HRQoL, for example by skin sparing techniques, should be assessed [43, 44]. To anticipate patients’ needs and perspectives, active involvement, open communication and adequate information is recommended. Regarding future studies, this study suggests that PROMs used for burn care research, may be appropriate to use in quantitative quality of life studies in NSTI survivors. For the more specific NSTI related consequences, regarding misdiagnosis, as well as fear for re-infection and actual re-infection, some additional questions could be included.

Strengths of this study include the adherence to the COREQ methodology, involvement of a patient representative and a patient group, and the combination of focus groups and individual interviews. Other strengths, contributing to validation of this study’s findings, are the use of different interviewers with different backgrounds, and triangulation of data during analysis. Limitations include the use of convenience sampling leading to possible overrepresentation of those more severely affected, as well as female overrepresentation compared to the literature. Other differences compared to the general population of patients with NSTI are the relatively young age, low number of comorbidities, high prevalence of affected anogenital and abdominal involvement, and absence of amputations in any of the participants [3, 14, 45]. This may limit the generalization of the results to all NSTI survivors. Furthermore, the extensive variation in the time since diagnosis may cause bias. In addition, a cluster analysis would have improved internal validation of the themes and subthemes. However, since the themes identified in this study could be validated by other studies, this underpins the validity of the results.

In conclusion, NSTI is a disease with a large impact on long-term physical and psychosocial wellbeing of the survivors and their relatives. From a medical point of view, more efforts are needed to improve recognition of the disease as well as surgical management. From a psychosocial point of view, screening and treatment for psychosocial consequences is recommended. Clinicians should also take time to reflect on the diagnostic phase and possible mistakes made. Furthermore, there is room for improvement regarding shared decision-making, care transitions, and understandable information should be made available to patients and their families. Last, the identified domains in this study may guide PROM selection for future quantitative HRQoL studies, which are needed to quantify the impact of disease.
